# Spatial evolution of microglial phenotypes and iron accumulation in CSF1R‐associated leukoencephalopathy

**DOI:** 10.1111/bpa.70157

**Published:** 2026-11-11

**Authors:** Julia Lier, Alireza Moein, Alonso Barrantes‐Freer, Dennis‐Dominik Rosmus, Markus Ponleitner, Jonas Franz, Ute Römuß, Evgeniya Kirilina, Annett Halle, Wolfgang Köhler, Peter Wieghofer, Joseph Classen, Ruth Stassart, Simon Hametner, Christine Stadelmann, Caroline G. Bergner

**Affiliations:** ^1^ Department of Neurology University Medical Center Leipzig Germany; ^2^ Paul‐Flechsig‐Institute of Neuropathology University Medical Center Leipzig Germany; ^3^ Institute of Anatomy Leipzig University Leipzig Germany; ^4^ Cellular Neuroanatomy Institute of Theoretical Medicine, Augsburg University Augsburg Germany; ^5^ Department of Neurology Medical University of Vienna; Comprehensive Center for Clinical Neurosciences and Mental Health Vienna Austria; ^6^ Department of Neuropathology University Medical Center Göttingen Göttingen Germany; ^7^ Department of Neurophysics Max Planck Institute for Human Cognitive and Brain Sciences Leipzig Germany; ^8^ German Center for Neurodegenerative Diseases (DZNE) Bonn Germany; ^9^ Division of Neuropathology and Neurochemistry, Department of Neurology and Comprehensive Center for Clinical Neurosciences and Mental Health Medical University of Vienna Vienna Austria

**Keywords:** CSF1R, leukodystrophy, microglia

## Abstract

Colony‐stimulating factor 1 receptor (CSF1R)‐associated leukoencephalopathy (CSF1R‐LE) is a fatal genetic white matter disease, whose onset is most commonly in mid‐age adulthood, and is characterized by rapid neurological decline and premature death within a few years after disease onset. CSF1R is exclusively expressed by microglia in the parenchyma of the adult central nervous system, thus defining CSF1R‐LE as a paradigmatic primary microgliopathy. However, the cellular alterations that drive the sudden onset and rapid progression of the disease remain largely unknown. Therefore, the investigation of microglia phenotypes in human histopathological tissue, particularly considering the spatial evolution of the pathology, can provide crucial insights into the disease‐causing mechanisms. In this study, we (immuno‐) histochemically characterized myeloid cell pathology in relation to the extent of damage to the axon‐myelin unit in five autopsy cases of CSF1R‐LE. A particular focus was on the relationship between microglia phenotypes and the pronounced iron accumulation observed in this disease. Our findings provide evidence that the early loss of homeostatic microglia and inflammatory activation of remaining myeloid cells are key components of areas with mild tissue pathology. Furthermore, we demonstrate a marked and progressive accumulation of iron within myeloid cells paralleling increasing white matter damage. This process is associated with ongoing myelin degeneration and occurs in the apparent absence of efficient remyelination. In summary, our study reveals stage‐specific myeloid cell pathology in CSF1R‐LE and highlights a specific spatial heterogeneity that should be considered when studying this disease. Moreover, our findings suggest that cellular iron accumulation may not only contribute to disease progression but may also represent a promising and clinically accessible biomarker for CSF1R‐LE.

## Graphical abstract

CSF1R‐LE autopsy tissue shows early loss of homeostatic microglia and inflammatory myeloid activation in mildly damaged regions, with progressive iron accumulation paralleling white matter damage and myelin degeneration without remyelination. This spatially heterogeneous pathology suggests cellular iron accumulation might accompany or even drive progression and serve as an accessible biomarker.

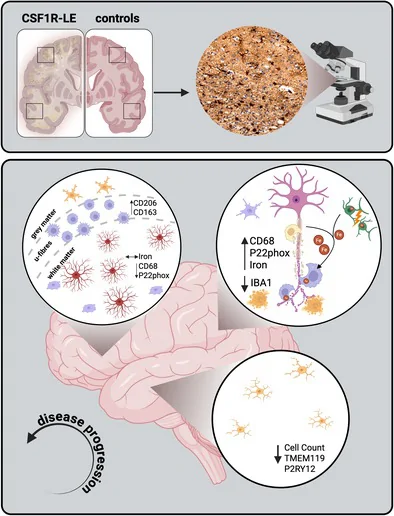

## 1 INTRODUCTION

Colony stimulating factor 1 receptor (CSF1R)‐associated leukoencephalopathy (CSF1R‐LE) is a rare, adult‐onset and invariably fatal genetic white matter disease characterized by progressive cognitive, behavioral, and motor decline. Mutation carriers usually remain asymptomatic for decades, but disease onset is typically followed by rapid clinical decline with an average survival of less than 7 years [1, 2]. The identification of pathogenic variants in the *CSF1R* gene as the underlying cause established CSF1R‐LE as a primary microgliopathy [3]. Emerging success of hematopoietic stem cell transplantation, which is thought to act—at least in part—through replacement of resident myeloid cells, further supports the central role of microglia in the disease onset and progression [4, 5, 6]. CSF1R is a receptor tyrosine kinase that, in the brain parenchyma, is exclusively expressed on microglia, where it mediates signaling pathways regulating cell motility, development, and survival ([7, 8]). Pathogenic variants cluster within the tyrosine kinase domain and presumably impair receptor activation [3]. Since CSF1R functions as a ligand‐induced dimer, dysfunctional monomers reduce active CSF1R signaling to approximately 25% of physiological levels [9].

However, the exact effects of pathogenic variants on microglia cells and how these variants lead to damage to the surrounding white matter are not yet sufficiently understood. Specifically, to what extent disease onset and progression is driven by a loss of homeostatic support from dysfunctional microglia versus the acquisition of deleterious, disease‐promoting phenotypes is unresolved. Animal models harboring human orthologous pathogenic variants in the *CSF1R* gene demonstrate reduced microglia density and mild white matter pathology [10, 11, 12]. Furthermore, mice with heterozygous loss of CSF1R recapitulate features of the disease including axonal degeneration, myelin abnormalities, and cognitive decline [7], and microglia have been proven to trigger pathology in the model [13, 14]. Though those models offer an opportunity to study important aspects of the pathology, it remains unclear if they can reproduce the full spectrum of the human disease. Specifically, the long asymptomatic phase followed by the abrupt and remarkably rapid clinical progression observed in patients has not yet been recapitulated. In light of this explanatory gap, the systematic examination of human brain tissue is essential to unravel the temporal sequence of pathological alterations and to clarify the cellular mechanisms driving disease pathogenesis.

Pigmentation within myeloid cells is a pathological hallmark of CSF1R‐LE that gave rise to its early histopathological designation as leukodystrophy with axonal spheroids and pigmented glia [15]. This pigmentation has been attributed to the accumulation of lipofuscin. In addition iron deposition was identified as a major contributor [16]. Iron metabolism has emerged as a critical modulator of myeloid cell function across a spectrum of neurological disorders, including multiple sclerosis [17, 18], neurodegenerative diseases [19], and cerebral ischemia [20]. In these contexts, iron actively shapes inflammatory responses and cell survival pathways [21]. Given the functional importance of intracellular iron load in myeloid cells, dysregulated iron handling represents a compelling candidate mechanism that may influence key pathogenic pathways in CSF1R‐LE. Furthermore, advances in iron‐sensitive magnetic resonance imaging techniques now allow in vivo assessment of iron redistribution, rendering iron a promising, clinically accessible imaging biomarker of disease activity [22, 23]. However, despite its early recognition as a histopathological feature, the spatial pattern of iron distribution in CSF1R‐LE remains largely unexplored—particularly with regard to its relationship to evolving microglial phenotypes and progressive tissue damage.

In the present study, we investigated myeloid cell phenotypes in correlation with the degree of white matter damage in postmortem brain tissue of patients with genetically confirmed CSF1R‐LE, with a particular focus on iron metabolism. By correlating iron accumulation with changes in myeloid cell density and polarization, as well as oligodendrocyte lineage pathology and regeneration across cortical and white matter regions, we aimed to gain insight into potential disease‐specific pathomechanisms. Furthermore, our study provides a rationale to further investigate iron distribution as a biologically meaningful substrate for the development of novel in vivo imaging biomarkers in CSF1R‐LE.

## 2 METHODS

### 2.1 Human tissue

Evolution of pathology in CSF1R‐LE was studied in formalin‐fixed, paraffin‐embedded (FFPE) brain tissue from five autopsy cases of patients with CSF1R‐LE and 15 control cases without confounding CNS pathology (Supplementary Table 1) obtained from the archives of the Myelin Brain Bank Leipzig, the DZNE Brain Bank, the Institute of Anatomy in Leipzig, and the Institute of Neuropathology at Medical University of Vienna. Patient data are summarized in Supplementary Table 1. Tissue blocks were obtained from frontal, parietal, temporal, occipital, and cerebellar lobes in CSF1R‐LE brains and frontal and occipital lobe and cerebellum in controls. The study was approved by the ethical review committees of the respective centers (Leipzig: 228/26, 375/22, 129/21; Bonn: 197/17; Vienna: 1067/2024) and written informed consent obtained from patients or legal representatives. All experiments were conducted in accordance with the Helsinki Declaration of 1975.

### 2.2 Case description

Case (#1) was a female patient with symptom onset at the age of 43. First symptoms included cognitive decline and behavioral abnormalities (lack of drive, compulsive buying). She developed impaired balance and motor deficits, causing the need for walking aids. Rapid progression of symptoms led to a vegetative state, resulting in death 3 years after symptom onset. Case (#2) was a male patient with first symptoms (memory deficits, behavioral changes) at the age of 49. Rapid progression occurred with disinhibited behavior, apraxia, and incontinence, spastic paraparesis, and pyramidal signs. The patient died 3 years after symptom onset due to severe pneumonia. Case (#3) was a female patient with symptom onset at the age of 60. Initial symptoms consisted of speech impairment and gait disturbance, followed by progressive cognitive decline. The patient died 6 years after symptom onset from an acute myocardial infarction. There was no information on the clinical presentation of case #4.

Case #5 was a male patient with symptom onset at the age of 51. First symptoms consisted of a personality change, which were followed by prolonged and generalized tonic–clonic seizures, non‐convulsive status epilepticus, and cognitive worsening characterized by generalized slowing, acalculia, aphasia, apraxia, and frontal symptoms together with dopamine‐unresponsive parkinsonism. Cranial MRI showed bilateral white matter hyperintensities on T2‐weighted images without Gadolinium enhancement, as well as callosal atrophy. The patient died from aspiration pneumonia at the age of 58. The case was previously published as a case report [24].

### 2.3 Histology and immunohistochemistry

For paraffin serial sections we performed hematoxylin and eosin (H&E) and luxol fast blue combined with periodic acid‐Schiff (LFB‐PAS) staining, as well as immunohistochemistry for a variety of microglia/macrophage and oligodendrocyte markers (see Supplementary Table 2). For immunohistochemistry, antigen retrieval was performed using a citrate buffer (95°C, 5 min). Then sections were pretreated with 1.5% H_2_O_2_ in methanol for 20 min to block endogenous peroxidase activity. To prevent nonspecific binding, tissue was blocked with 1% bovine serum albumin (BSA, Sigma‐Aldrich, A7906) in PBS/Triton (0.03%, PBS‐T). Subsequently, primary antibodies were incubated overnight at 4°C diluted in 0.5% BSA in PBS‐T (Supplementary Table 2). After rinsing with PBS‐T, the biotinylated secondary antibodies (1:100) were applied and incubated for 90 min at room temperature, followed by avidin‐conjugated peroxidase incubation (extrAvidin‐Peroxidase, Sigma‐Aldrich E2886; 1:100) and visualization by 3,3′ diaminobenzidine tetrahydrochloride (DAB, Sigma‐Aldrich D5905) as a chromogen. After counterstaining using hematoxylin, sections were washed, dehydrated and coverslipped. Perls' iron staining was performed on tissue sections, which were incubated for 2 h at 37°C in equal parts (1:1) 5% potassium hexacyanoferrate(II) and 5% hydrochloric acid. Sections were subsequently washed in PBS and TRIS–HCl buffer (pH 8.0) and chromogenic signal was developed using DAB as the chromogen. After counterstaining with hematoxylin, sections were washed, dehydrated, and mounted. For double staining, following DAB development, sections were incubated with a primary antibody as described above. After rinsing, alkaline phosphatase–conjugated secondary antibodies were applied, and staining was developed using the Vector Red detection system (Vector, SK‐5100). Sections were then counterstained with hematoxylin, washed, dehydrated, and mounted. For Neurofilament (NF), Myelin Basic Protein (MBP), Tubulin Polymerization Promoting Protein (TPPP) automated immunohistochemistry was performed using the Benchmark Ultra Staining System (Roche).

For fluorescence imaging, primary antibodies diluted in 0.5% BSA in PBS/Triton were applied overnight at 4°C, followed by washes and incubation with fluorophore‐conjugated secondary antibodies (Supplementary Table 2) for 60 minutes at room temperature in the dark. After additional washes, nuclei were counterstained with 4′,6‐Diamidino‐2‐phenylindole (DAPI), rinsed in PBS and distilled water, treated with TrueBlack Autofluorescence Quencher (Biotium, Cat.‐Nr. 23,007) to reduce autofluorescence, and mounted with Dako fluorescence mounting medium (Cat.‐Nr. S3023) for imaging.

### 2.4 Image analysis

Slides were fully digitalized using digital slide scanners (Pannoramic Scan II, 3D HISTECH Ltd., Budapest, Hungary; Zeiss Axioscan 7, Carl Zeiss Microscopy GmbH, Jena, Germany) at 10x magnification. Image Analysis was performed using Qupath (Version 0.5.1, [25]), ImageJ (Version 1.54 g, [26]) and OMERO (Version 5.24.0, [27]). Lesion staging and annotation was based on MBP, amyloid precursor protein (APP), Glial fibrillary acidic protein (GFAP) and LFB‐PAS staining as described below. Mean cell count per mm^2^ for each scan was quantified by random sampling of 500 × 500 μm regions of interest across all lesion stages. Group means were calculated from the per‐patient mean values. Analysis of Nearest Neighbor Distance was achieved using manually distributed annotations (3 × 3mm) in the grey matter of IBA1 stained scans. Separating color channels was accomplished using the “Colour Deconvolution” plugin for ImageJ (1.51r). Thresholding was performed in ImageJ and controlled manually for misdetections, for example, of vessels or tissue wrinkles. Images were converted to masks, then particle distribution function was used to detect particles >10 μm^2^ and median nearest neighbor distance (biovoxxel plugin [28]) was calculated.

### 2.5 Statistical analysis

All analyses were performed in the R programming environment. Given the small cohort size (*n* = 5 patients), we deliberately refrained from inferential statistical testing for most comparisons and restricted our analysis to descriptive statistics; cell densities are reported as mean ± SEM, calculated from patient‐level means. Formal statistical testing was applied only to nearest neighbor analysis. For this analysis, data were first assessed for normality using the Shapiro–Wilk test. Non‐parametric comparisons were analyzed using the Mann–Whitney U test. The level of statistical significance was defined as α = 0.05.

## 3 RESULTS

### 3.1 Classification of histopathologic stages in CSF1R‐LE White matter

To classify the evolution of white matter lesions, (immuno)histochemical stainings were performed on tissue blocks obtained from the frontal, parietal, temporal (including hippocampal), and occipital lobes, as well as from the cerebellum. Lesion staging was based on myelin lipid staining (LFB–PAS) and immunohistochemistry for MBP to assess myelin integrity, NF to evaluate axonal density, APP as a marker of acute axonal injury, and GFAP to determine the extent of astrocytic scar formation. Four lesion stages were distinguished: (I) normal‐appearing white matter, characterized by preserved myelin and absence of axonal injury; (II) early lesions, defined by the presence of scattered axonal spheroids without apparent loss of myelin or neurofilament density; (III) progressive lesions, showing reduced NF‐positive axonal density, diminished myelin, and the presence of APP‐positive spheroids; and (IV) scar tissue, marked by severe loss of myelin and axons and prominent astrocytic scar formation (Figure 1, Supplementary Table 3). Across all cases, frontal white matter exhibited the most advanced pathology (stage IV). In contrast, occipital regions were only mildly affected (Stages I–II) and, together with temporal brain areas (Stage I), represented the best‐preserved neopallial regions. Parietal areas presented with intermittent to advanced tissue damage (Stage II to IV).

**FIGURE 1:**
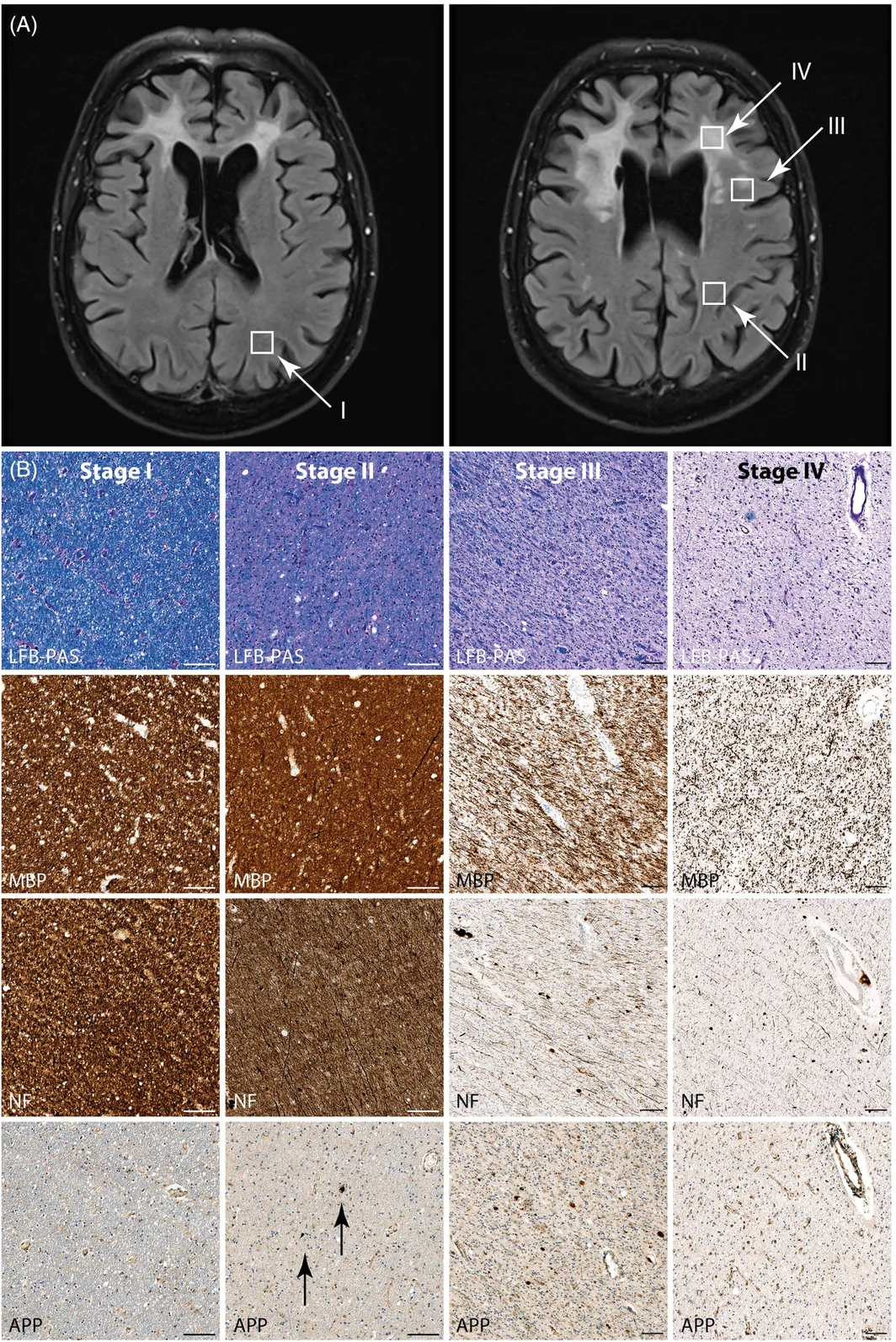
Staging of lesion pathology in CSF1R‐LE. (A) Exemplary FLAIR images from last pre‐mortem MRI of Case #2, with predominant frontal affection and relative preservation of occipital areas. Regions corresponding to the respective postmortem lesion stages are indicated. (B) Exemplary images of myelin lipid (LFB‐PAS), myelin bprotein (MBP), axon density (NF), and axon damage (APP) staining with stage specific increase in pathology as detailed in Supplementary Table 3. Scale bar: 100 μm.

### 3.2 Microglial loss as an early pathological feature in CSF1R‐LE

Next, we assessed the densities and spatial distribution of white matter myeloid cells using a comprehensive panel of markers capturing microglia and other myeloid cells across homeostatic and activated states (Supplementary Table 2). Temporal regions (Stage I) showed the best preservation of myeloid cell phenotypes, with mildly activated yet predominantly branched morphology (Figure 2B). In occipital regions (Stage I–II), microglia were activated, as indicated by increased expression of CD68 and an amoeboid shape. While microglia density was only mildly reduced in temporal regions (74.1 ± 11.5 IBA1^+^ cells/mm^2^ versus 127.7 ± 15.3 cells/mm^2^ in control tissue, Supplementary Figure 1), occipital regions displayed extended areas with markedly reduced microglia numbers (33.1 ± 4.1 IBA1^+^ cells/mm^2^ versus 136.5 ± 16.8 cells/mm^2^ in control tissue). Spots of elevated density were observed, often surrounding blood vessels (Figure 2C, arrows). With progression of tissue damage (Stage II‐III) despite ongoing overall reduction in IBA1^+^ myeloid cells (stage II: 49.5 ± 10.6; stage III: 37.8 ± 8.1 IBA1^+^ cells/mm^2^), regions with intense decay of the axon‐myelin units were accompanied by focal accumulations of foamy, CD68^+^ phagocytes (Figure 2D, arrow). Correspondingly in stage III, an increase in CD68^+^ cells correlated with elevated axonal spheroid densities (Figure 2H–J). In progressed scar tissue (frontal regions, Stage IV), phagocyte density was severely reduced (16.1 ± 4.6 IBA1^+^ cells/mm^2^ versus 125.8 ± 8.9 cells/mm^2^ in control tissue), with most of the cells residing within subcortical U‐fibers, where myelin was comparatively better preserved (Figure 2E,F). While CD68 expression was increased on remaining myeloid cells compared to control tissue (Figure 2I), markers of a homeostatic microglial phenotype were largely absent from white matter lesions, except for hippocampal regions and the cerebellum, where sparse TMEM119‐positive expression was detected on IBA1^+^ cells (Figure 2K). Compared with the white matter, neocortex showed better preservation of myeloid cells, where microglia exhibited a predominantly branched albeit frequently dystrophic morphology (Figure 2L, inset). In most regions, cortical microglial densities were reduced, with supragranular layers often being relatively spared (Figure 2M). Clusters of myeloid cells were observed within cortical regions surrounding areas classified as Stages II to IV (Figure 2L). Consistent with the visual impression of clustering, nearest‐neighbor analysis revealed a marked reduction in nearest neighbor distance (Δ = −10), indicating a strong deviation from a random spatial distribution (control grey matter: Δ = +0.6, *p*< 0.001, Supplementary Figure 2A, B). Clustered myeloid cells showed expression of IBA1 and the homeostatic microglial marker P2RY12, alongside low levels of CD68 and FTL, indicative of a non‐activated state. Notably, TMEM119 expression was absent in this cluster (Supplementary Figure 2C–K).

**FIGURE 2:**
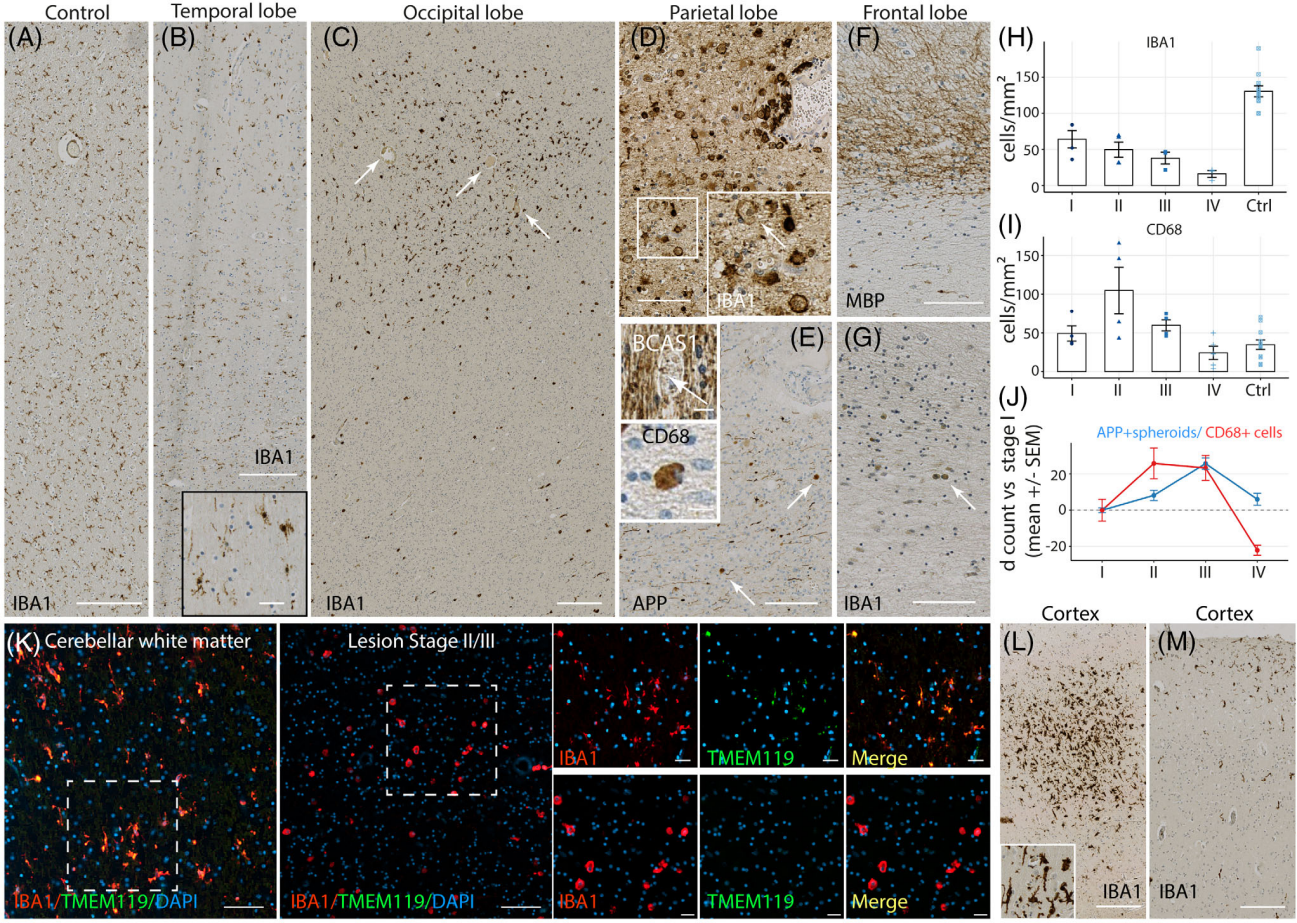
Evolution of myeloid cell density in CSF1R‐LE stages. (A) IBA1^+^ microglial density in control white matter. (B) In temporal lobe tissue, microglial density is only mildly reduced. (C) In the occipital lobe (Stage I), myeloid cell density is decreased, with focal perivascular accumulations of myeloid cells (arrows on vessels). (D) In Stage II/III lesion areas, microglia show downregulation of IBA1 expression (inset, arrow). (E) APP immunostaining on a serial section demonstrates axonal injury and ongoing myelin phagocytosis (arrows on exemplary APP+ spheroids), insets show exemplary myeloid cells in Stage III with evidence of myelin ingestion (arrow in BCAS1 staining indicates intracellular myelin debris) and intense expression of CD68. (F) In advanced Stage IV pathology, myeloid cells are largely restricted to subcortical regions, corresponding to areas of residual myelin (G). (H), (I) Quantification of myeloid cell densities across lesion stages. Values are given as mean ± SEM of cells/mm^2^, calculated from patient‐level means. (J) The density of APP‐positive spheroids corresponds to increase in CD68^+^ myeloid cells at lesion Stage III. (K) Restricted expression of the homeostatic microglial marker TMEM119 on IBA1^+^ myeloid cells in the cerebellum. (L) Focal clusters of IBA1^+^ microglia are observed in cortical tissue, and cells are relatively preserved in supragranular layers (M). Scale bars: B, C, L, M 250 μm, D‐G 100 μm, insets in E 10 μm, B, D 50 μm. K 50 μm, enlarged images 20 μm.

### 3.3 Progressive iron accumulation in myeloid cells during white matter lesion development

We analyzed the distribution of iron in CSF1R‐LE brain tissue across the different lesion stages. Histochemical detection using Perls' Prussian blue staining in Stage I lesion areas (occipital and temporal lobe) revealed iron localization resembling that of control white matter, with staining of myelin sheaths and enrichment within oligodendrocyte cell bodies in both white matter and cortex (Figure 3A,C). Double labeling with IBA1 in these areas showed that ramified microglia in both white and gray matter were only rarely iron‐positive (5.2 ± 1.2 cells/mm^2^ in white matter areas, Figure 3C,D). In contrast, amoeboid myeloid cells in later lesion stages demonstrated marked iron accumulation, seemingly corresponding to the progression of lesion pathology (46.8 ± 12.5 in Stage II and 55.9 ± 10.8 cells/mm^2^ in Stage III areas, Figures 3E–G and 4B). Corresponding to myelin loss, iron staining of myelin sheath progressively diminished. Oligodendrocytes in damaged areas surrounding amoeboid cells likewise exhibited reduced iron content (Figure 3G,I). Interestingly, within perivascular myeloid cell clusters at stage II and early phases of stage III cells often were iron negative in the immediate vicinity of the vessel (Figure 3H,I). In advanced lesion Stage IV, iron in myelin sheaths was concentrated within subcortical U‐fibers. Here, most myeloid cells showed intense iron accumulation (Figure 3J–M).

**FIGURE 3:**
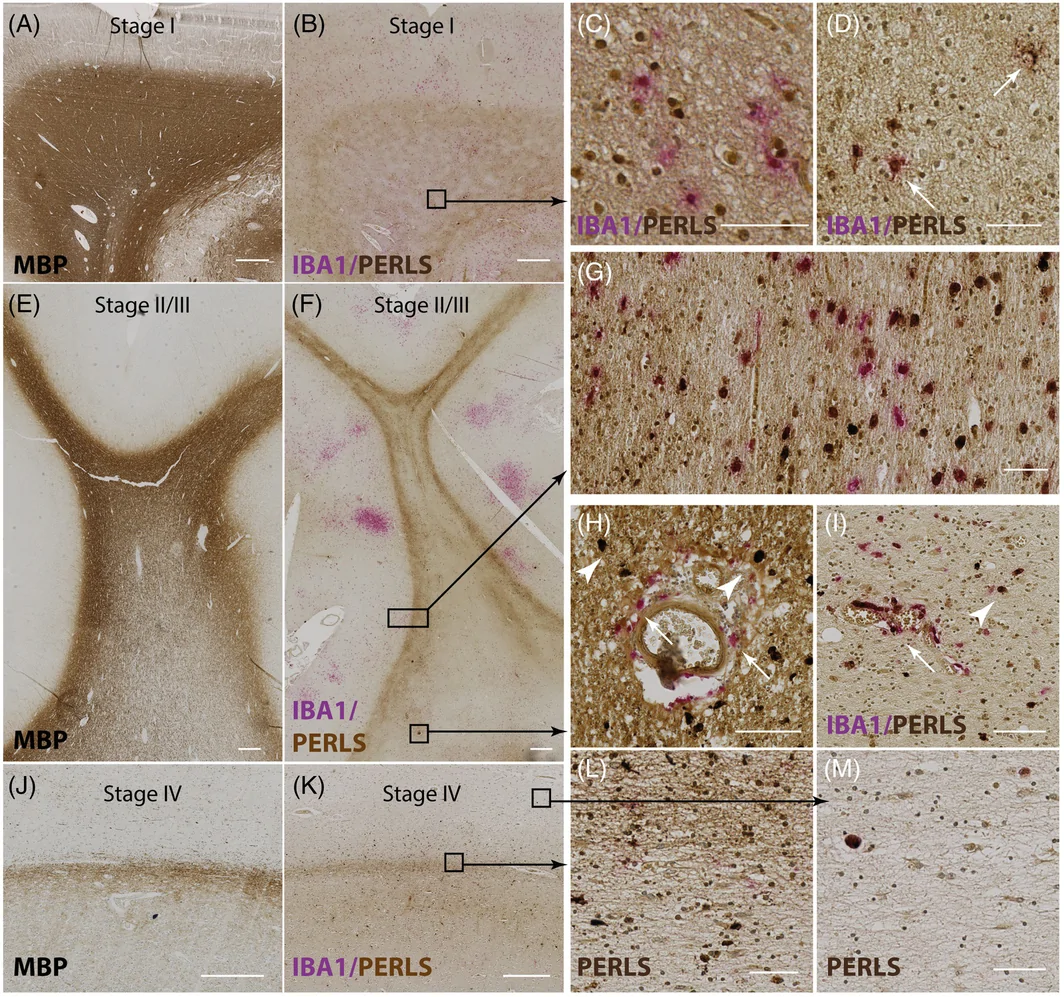
Iron distribution across lesion stages. (A) Myelin density in Stage I lesions assessed by MBP immunohistochemistry. (B) Perls' staining for iron on a serial section demonstrates iron enrichment within compact myelin and oligodendrocytes. Double staining for IBA1 and iron reveals predominantly iron‐negative (C) and few branched iron‐positive myeloid cells in early Stage I lesions (D, arrows). (E) Stage II/III lesion areas show ongoing myelin loss, accompanied by focal cortical clusters of IBA1^+^ cells (F) and increasing numbers of amoeboid, intensely iron‐positive myeloid cells in the white matter (G). (H), (I) Perivascular IBA1^+^ cells (arrows) display low iron content compared to iron‐rich parenchymal myeloid cells (arrowheads). (J), (K) In advanced Stage IV lesions, residual myelin and myeloid cells are enriched in the subcortical U‐fibers. (L), (M) Myeloid cells in the subcortical U‐fibers, as well as sparse myeloid cells in the lesion scar center, are iron‐rich. Scale bar: A, B, E, F, J, K: 500 μm; C, D, G, H, I, L, M: 50 μm.

**FIGURE 4:**
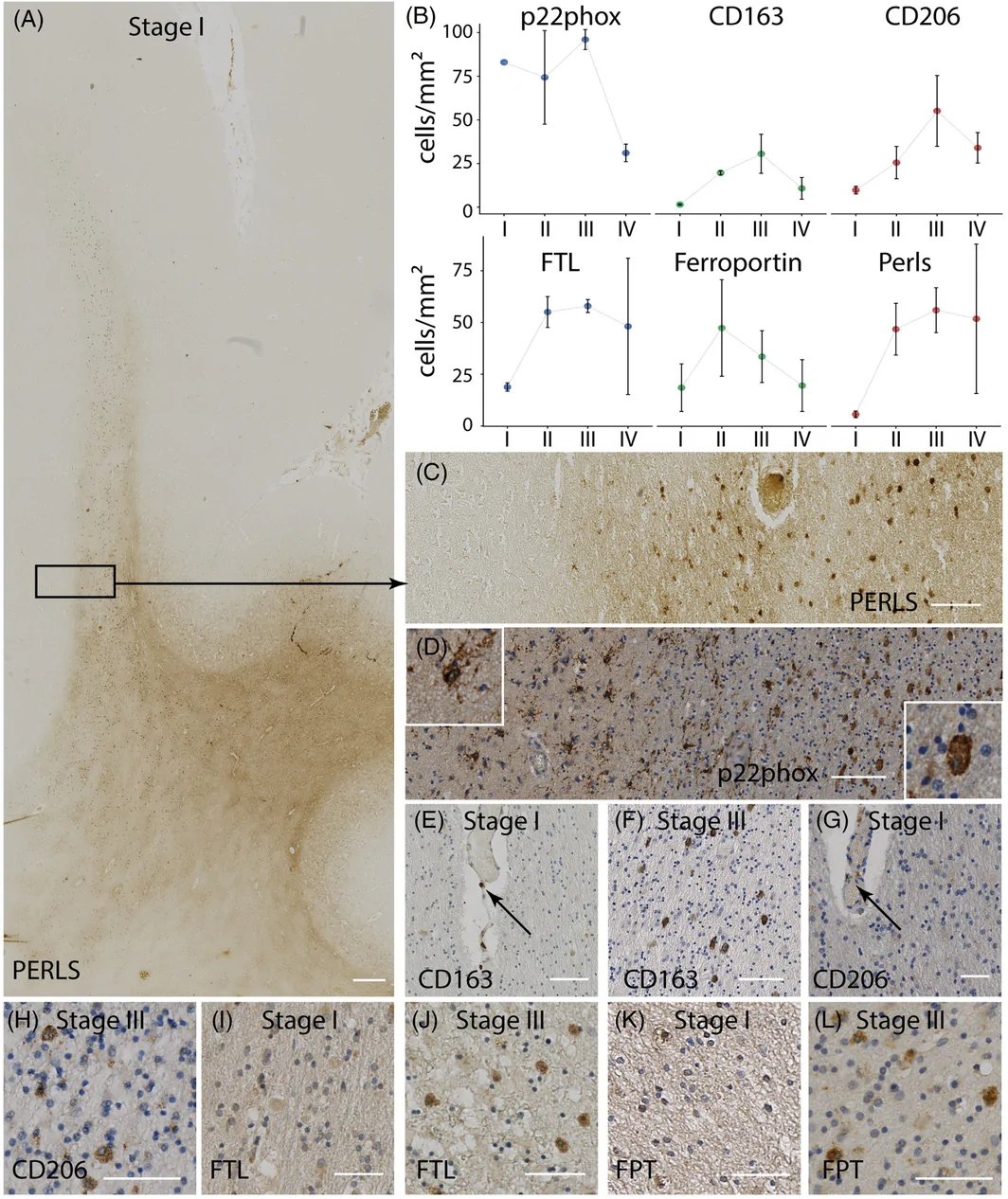
Patterns of myeloid cell polarization across lesion stages. (A) Histochemical detection of iron in occipital lobe tissue (Stage I). (B) Quantification of myeloid activation markers and iron metabolism‐related proteins across lesion stages. Values are given as mean ± SEM of cells/mm^2^, calculated from patient‐level means (*n* = 5). (C), (D) Iron‐positive macrophages in the white matter and microglia in the adjacent gray matter on serial sections express p22phox. (E), (F) CD163, (G), (H) CD206, (I), (J) Ferritin light chain (FTL), and (K), (L) Ferroportin (FPT) expression in early and advanced lesion stages. Arrows indicate characteristic CD206^+^ and CD163^+^ perivascular macrophages in early disease stages. Scale bar: A: 500 μm, C, D, G, H: 100 μm, E‐F, I‐L: 50 μm.

### 3.4 Early activation of ROS pathways in myeloid cells in CSF1R‐LE

The membrane‐associated enzyme phagocyte NADPH oxidase (p22phox) is a key component in the generation of reactive oxygen species (ROS) during inflammatory activation of myeloid cells. P22phox expression was increased in myeloid cells from early stages of pathology (83 ± 6.0 p22phox^+^ cells in Stage I white matter, Figure 4B) including a subset of ramified microglia, and gray matter microglia cells in adjacent cortical regions (Figure 4A,D). Densities of amoeboid p22phox^+^ cells remained high throughout Stages II and III (74.3 ± 26.8 cells/mm^2^ in Stage II, 95.9 ± 5.7 cells/mm^2^ in Stage III, Figure 4B) with reduction only toward scar tissue (31.1 ± 5.0 cells/mm^2^). In contrast, CD163 was expressed on few amoeboid cells in lesion Stage I (1.5 ± 0.6 cells/mm^2^, Figure 4B,E) with increase in CD163^+^ myeloid cell density only from Stages II–IV (Figure 4B,F, 19.7 ± 1.2 cells/mm^2^ in Stage II, 30.5 ± 11.2 cells/mm^2^ in Stage III, and 10.7 ± 6.2 cells/mm^2^ in Stage IV). To further characterize myeloid activation states, we examined mannose‐receptor (CD206) expressing, alternatively activated macrophages. CD206^+^ cells followed a similar spatial pattern as CD163 with increasing expression in the more advanced stages of tissue damage (9.7 ± 2.2 cells/mm^2^ in Stage I, 25.5 ± 9.2 cells/mm^2^ Stage II, 55.1 ± 20.3 cells/mm^2^ in Stage III, 34.0 ± 8.7 cells/mm^2^ in Stage IV Figure 4B,G,H). Next, we correlated inflammatory marker expression to immunohistochemical markers of iron metabolism. Ferritin light chain, indicative of intracellular iron storage, increased in parallel with stage‐dependent iron accumulation in myeloid cells (18.2 ± 2.0 cells/mm^2^ in Stage I vs. 57.9 ± 3.1 cells/mm^2^ in Stage III, Figure 4B,I,J). In contrast, Ferroportin, a marker of active iron export, was prominently expressed on non‐myeloid cells, that is, oligodendrocytes and astrocytes, in early lesion stages (Stage I Figure 4K), and showed increased expression on macrophages in areas with more advanced tissue damage (18.5 ± 11.5 cells/mm^2^ in Stage I, 47.3 ± 23.3 cells/mm^2^ in Stage II, 33.5 ± 12.5 cells/mm^2^ in Stage III, Figure 4B,L).

### 3.5 Lack of remyelination in CSF1R‐LE

Lesion stages were defined by the progressive loss of axons and compact myelin assessed by staining of myelin sheath components (MBP, LFB). Consistent with myelin demise, quantification of mature oligodendrocyte cell bodies in CSF1R‐LE revealed a stage‐dependent decline in TPPP^+^ cells. Mean cell densities were highest in early lesion stages (546.5 ± 65.8 cells/mm^2^ in Stage I, 495.3 ± 65.3 cells/mm^2^ in Stage II, Figure 5A) and decreased markedly in more advanced stages (198.5 ± 92.2 cells/mm^2^ in Stage III, 48.7 ± 32.7 cells/mm^2^ in Stage IV, Figure 5B,C,F). To assess potential remyelination, we utilized BCAS1 as a marker of oligodendrocytes engaged in active myelin formation during development and regeneration. With progressive tissue destruction, BCAS1 staining in compact myelin was lost in parallel with MBP. Although occasional cells upregulated BCAS1 expression in damaged regions, no ramified, remyelinating oligodendrocyte phenotypes were detected in white matter at any lesion stage (qualitative observation; Figure 5D,E).

**FIGURE 5:**
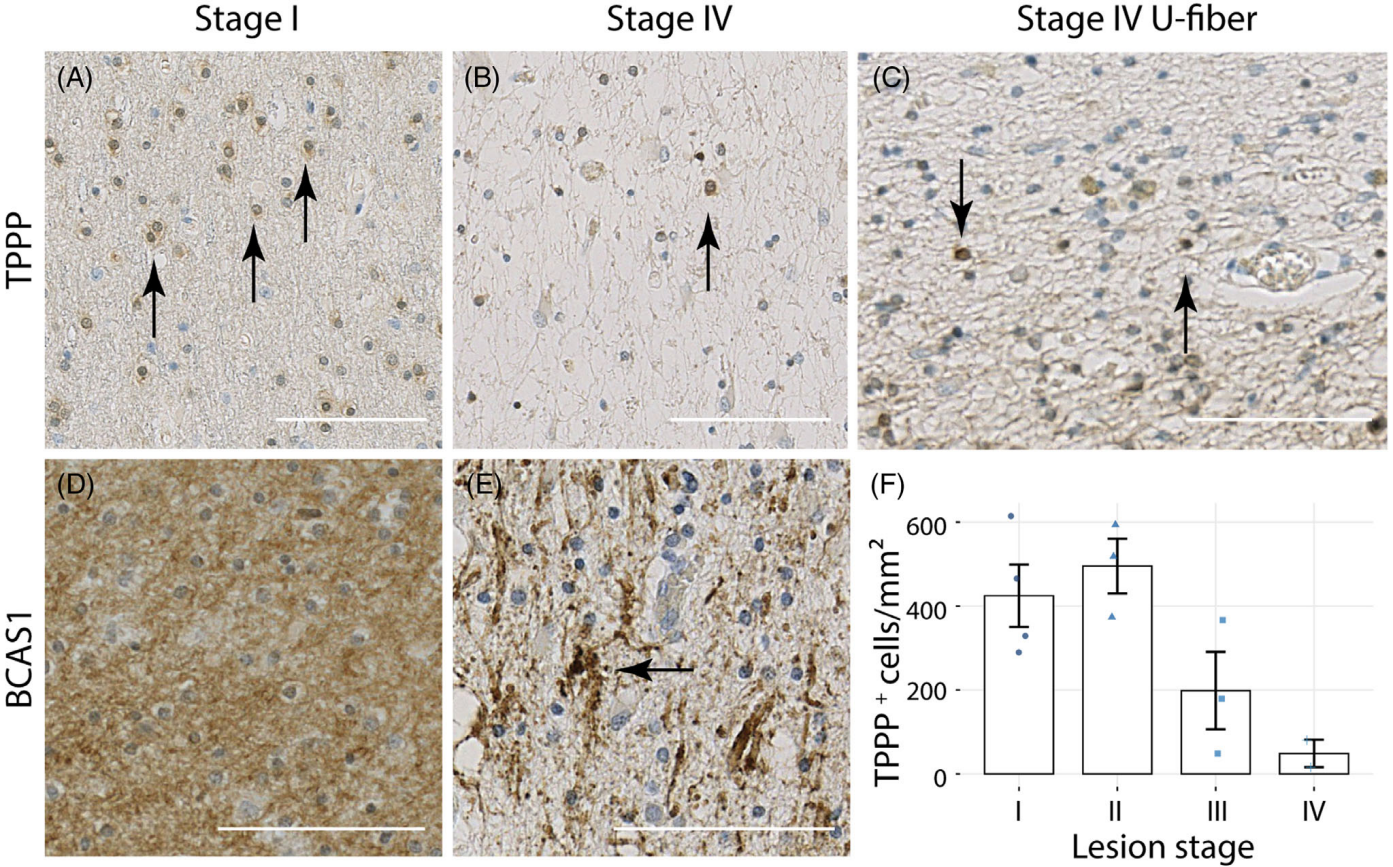
Oligodendrocyte loss with no evidence of active remyelination. (A), (B) TPPP/p25^+^ cell densities in early lesion stages compared with severely reduced numbers of TPPP^+^ cell bodies in advanced tissue (arrows indicate TPPP^+^ cell bodies). (C) In subcortical U‐fibers at late pathological stages, increased but partly pyknotic oligodendrocyte cell bodies are observed (arrowheads). (D) BCAS1 shows staining of compact myelin in early stages. (E) In advanced pathology, oligodendrocyte cell bodies are partly intensely BCAS1^+^ (arrow), but ramified, actively remyelinating cells are absent from the tissue. (F) Quantification of TPPP^+^ oligodendrocytes across lesion stages. Values are given as mean ± SEM of cells/mm^2^, calculated from patient‐level means. Scale bar: 100 μm.

## 4 DISCUSSION

CSF1R‐LE is a fatal, adult‐onset white matter disease caused by pathogenic variants in the *CSF1R* gene. In the adult central nervous system, CSF1R expression is largely restricted to microglia [29] that have been implicated as key drivers of pathology [13]. However, the nature of early microglial alterations and the mechanisms that trigger disease onset as well as those that drive progression are only beginning to be understood. A detailed characterization of disease stage‐specific myeloid cell pathology is critical, both for improving early detection of disease onset and for guiding the development of myeloid‐directed therapeutic strategies. In this study, we performed an (immuno‐)histopathological analysis of postmortem cortical and white matter tissue from five genetically confirmed CSF1R‐LE cases and controls, encompassing areas with minimal structural damage as well as regions showing advanced axonal and myelin loss. Across patients, we observed a spectrum of tissue alterations following a consistent spatial pattern, ranging from regions with largely preserved axon–myelin architecture to areas of advanced astroglial scarring devoid of myelin and axons. As our analysis is based on post‐mortem tissue, this gradient cannot directly establish a temporal sequence; moreover, regional heterogeneity may reflect intrinsic differences in vulnerability rather than disease stage. Nevertheless, the gradual transitions and consistency of this pattern across patients are compatible with progressive tissue damage following a disease‐specific sequence.

The initial observation with regard to myeloid cells was a marked reduction in overall parenchymal microglial density. Our histopathological findings therefore suggested that early loss of homeostatic microglial functions may represent a key initiating event in CSF1R‐LE. This concept is supported by recent experimental animal models that have explored the consequences of long‐term microglial depletion. In *Csf1r* ^ΔFIRE/ΔFIRE^ mice, genetic deletion of an intronic superenhancer element of *Csf1r* leads to permanent depletion of endogenous tissue macrophages in selective organs, including the brain [30]. Although these models fail to reproduce the abrupt clinical onset observed in human disease, affected mice develop age‐dependent white matter pathology with similarities to CSF1R‐LE, including myelin abnormalities, axonal spheroids, and calcifications. Importantly, these changes can be partially reversed following microglial repopulation, underscoring the critical role of microglia in white matter maintenance [31, 32, 33].

In human tissue, signs of increased numbers of activated amoeboid and foamy myeloid cells become apparent in areas with progressed tissue damage. Distinguishing resident microglia from infiltrating monocyte‐derived macrophages based on marker expression remains challenging. This holds true particularly when homeostatic markers such as TMEM119, SALL1, and P2RY12 are downregulated on endogenous microglia—a phenomenon previously described in CSF1R‐LE [34] and confirmed in our cohort. While determination of peripheral origin by immunophenotyping is therefore limited, the accumulation of myeloid cells in perivascular niches and their patchy increase in the vicinity of blood vessels, especially in areas of advanced tissue destruction, suggests—though not proofs—that microglial loss may be accompanied by partial compensatory recruitment of peripheral myeloid cells. A prominent feature of the myeloid pathology in our study was the early and sustained upregulation of p22phox, indicating activation of NADPH complex and reactive oxygen species production. This was detectable already in sparse, ramified microglia at lesion stage I and cortical microglia [35, 36, 37]. Only in more advanced regions characterized by pronounced myelin degradation, phagocytic activity, and foam cell formation, we observed anti‐inflammatory and regenerative myeloid markers CD163 and CD206 on myeloid subsets and among p22phox expressing cells. This finding suggests early pro‐inflammatory activation of residual cells during initial tissue damage only slowly transitioning to dampening myeloid profiles with progression of phagocytic tissue removal, as has been described in myeloid cells under conditions of prolonged myelin uptake [38, 39, 40].

Pigmented myeloid cells represent a defining histopathological feature of CSF1R‐LE. Accumulation of autofluorescent lipofuscin has been proven [41, 42] with ceroid‐like multilamellar or fingerprint bodies as correlates at the ultrastructural level [43]. Notably, already the earliest histopathological reports by Van Borgart and Nissen documented increased iron deposition in myeloid cells in the disease, confirmed by later follow‐up studies [15, 16, 42, 44, 45, 46].

In our cohort, extensive iron deposition was detectable in all examined cases from the earliest lesional stages in activated myeloid cells increasing with progressive tissue destruction. Spatially, iron‐rich myeloid cells in early areas of active myelin decay were more abundant within deeper lesional parenchyma than in vessel‐adjacent perivascular myeloid cells. This argues against intravascular heme as the primary iron source, despite a substantial upregulation of the hemoglobin‐haptoglobin scavenger receptor CD163 in advanced lesion stages [22]. Instead, the concomitant loss of oligodendrocytes and myelin suggests a shift of iron from degenerating oligodendroglial lineage cells and myelin sheaths [18, 47].

Notably, we found no evidence of ongoing remyelination, as assessed by qualitative observation of BCAS1 expression, a well‐established marker of actively remyelinating oligodendrocyte lineage cells during development and regeneration [48, 49]. Recently, a single nucleus sequencing study in human postmortem tissue in CSF1R‐LE suggested a relative expansion of immature oligodendrocytes. Importantly, however, the absence of oligodendrocyte developmental stages associated with active myelin formation suggests that such a potential expansion is unlikely to result in the restoration of functional myelin sheaths. This apparent lack of remyelination occurs despite inflammatory activation of white matter myeloid cells, whose secreted mediators have previously been implicated in promoting the differentiation of oligodendrocyte precursor cells [50]. Iron‐rich pro‐inflammatory myeloid cells are a known feature of slowly expanding lesions in multiple sclerosis that are characterized by a lack of remyelination [51, 52]. Conversely, myeloid cells in remyelinated areas are typically iron‐poor [53], and anti‐inflammatory myeloid cells have been associated with increased expression of Ferroportin that may support iron export and fueling of regenerating OPCs [54, 55]. Thus, persistent iron sequestration within myeloid cells may correlate with irreversible tissue damage preventing iron recycling during myelin regeneration in CSF1R‐LE. In addition, CSF1R‐LE does not feature selective demyelination, but decay of the entire axon‐myelin unit is observed. The absence of effective long‐term remyelination, thus, in addition, is hindered by extensive axonal loss, as denuded axons can no longer serve as scaffolds for myelin regeneration [56].

Beyond their pathophysiological relevance, iron‐related changes may have implications for in vivo disease monitoring. Iron‐sensitive MRI techniques, such as quantitative susceptibility mapping, have proven valuable in assessing lesion activity in other neurologic disorders, for instance in slowly progressing multiple sclerosis [51, 57, 58]. Based on the histopathological distribution of iron observed here, we propose that similar approaches might detect iron redistribution in CSF1R‐LE, with loss from compact myelin and increased focal signal of myeloid cells in regions of active tissue damage dominated by iron‐laden phagocytes. In later stages, prominent signals from subcortical U‐fibers and reduced signal in fully scarred areas might be expected. Whether iron‐sensitive imaging can serve as a biomarker for disease onset and progression in CSF1R‐LE remains to be tested in prospective in vivo studies.

One intriguing finding of our study was the presence of marked region‐specific differences in tissue pathology and microglial preservation. These findings are reminiscent of recent studies highlighting regional differences in microglial density, morphology, and gene expression profiles [59] that likely contribute to functional specifications. Temporal—and to a lesser extent occipital—regions showed a surprising preservation of ramified or only mildly activated white matter microglia when compared with frontal and parietal areas. Similar patterns, including relative temporal sparing, have been reported in previous histopathological and neuroimaging studies [60, 61, 62, 63, 64]. In the cortex, microglia were generally better preserved than in the adjacent white matter, particularly within superficial cortical layers. In chronically damaged regions, we observed a relative persistence of amoeboid, phagocytic myeloid cells associated with residual myelin within subcortical U‐fibers. Potential mechanisms underlying regional vulnerability may include local differences in trophic factor availability, particularly ligands of the CSF1R pathway. In the murine brain, IL‐34 and CSF‐1 show largely non‐overlapping expression patterns, with IL‐34 predominantly expressed in gray matter—especially in upper cortical layers—whereas CSF‐1 is more prominent in white matter areas [65, 66]. The relative preservation of subcortical U‐fibers constitutes a metabolic pattern of white matter pathology and mirrors observations in other leukodystrophies but not multiple sclerosis. It may relate to late myelination, distinct metabolic demands, or differences in vascular supply, although the precise mechanisms remain incompletely understood and warrant further investigations.

Novel (multi‐)omic phenotyping approaches provide a more comprehensive view of myeloid phenotypes and necessitate a shift from traditional dichotomous classification into pro‐ and anti‐inflammatory states toward a nuanced framework capturing diverse and complex microglial activation states [67, 68]. Recent single‐nucleus transcriptomic studies in CSF1R‐LE brain tissue have accordingly identified disease‐specific signatures of activated myeloid cells, including increased oxidative stress responses, altered iron homeostasis signatures, and enrichment of ferroptosis‐related pathways [69, 70]. Interestingly and in those lines, a recent study in the haploinsufficient model of the disease was able to confirm transcriptional alterations of metal ion homeostasis, leading to intracellular iron accumulation, increased oxidative stress in microglia causing damage to surrounding neurons and oligodendrocytes [71]. This study highlights an important role of CSF1R in microglial ion homeostasis. Overall, these molecular findings closely align with our histopathological observation and suggest, in addition, that ferroptosis may contribute to the long‐term reduction of myeloid cell density in affected regions and that iron might trigger the production of detrimental myeloid‐derived reactive oxygen species. While single‐nucleus studies provide important insights into myeloid cell state complexity, our data reveal substantial spatial heterogeneity of microglial phenotypes and region‐specific microglial vulnerability. In addition, focal hotspots of tissue destruction and myeloid cell expansion or invasion are likely to generate a heterogeneous microscale landscape of pathological myeloid phenotypes, rendering spatial information an indispensable component to be incorporated in future studies. Due to the rarity of the disorder investigated, a principal limitation of our study is the relatively small sample size. Furthermore, all included patients exhibited a predominant lesion burden in the frontal lobe, whereas disease onset in the parietal white matter has been associated with primary motor symptoms ([1, 5]). The identified myeloid phenotypes and their association with progressive tissue pathology were remarkably consistent across all patients examined. We therefore consider these findings likely to reflect a generalizable sequence of tissue injury, although the spatial manifestation of this sequence may vary slightly depending on the predominant clinical phenotype.

## 5 CONCLUSION

In conclusion, our histopathological analyses reveal distinct myeloid phenotypes in CSF1R‐LE correlating with progression, axonal and myelin degeneration. Areas with minor axon‐myelin pathology are characterized by a loss of homeostatic microglia, while regions with progressed tissue decay are characterized by the predominance of activated myeloid cells with largely pro‐inflammatory and iron‐rich phenotypes. This predominant phenotype and the persistently reduced myeloid cell densities mirror key pathological features of smoldering lesions in multiple sclerosis. Importantly, these similarities do not necessarily imply shared initiating mechanisms but may instead reflect overlapping impairments in the clearance of damaged tissue, accompanied by comparable kinetics of myeloid cell turnover and persistent sequestration of myelin‐derived iron within myeloid cells during disease progression. The increasing availability of advanced ‐omics technologies offers powerful new opportunities to dissect disease mechanisms and to refine the phenotypic characterization of myeloid cell states in human pathology. Future studies should integrate high‐resolution transcriptomic profiling with spatially resolved approaches in *post‐mortem* tissue, linking distinct myeloid phenotypes to specific stages in the evolution of pathology and regional differences in myeloid cell vulnerability. Such spatial transcriptomic analyses may ultimately help to disentangle pathways driving disease initiation from those sustaining chronic progression, thereby informing more targeted therapeutic strategies.

## AUTHOR CONTRIBUTIONS

J.L. performed image and statistical analyses and assisted in preparing the first draft of the manuscript. A.M. conducted digital image acquisition. A.B.F. and R.S. performed immunohistochemical staining, provided neuropathological expertise, and contributed biological samples. D.D.R. and P.W. carried out immunofluorescence staining. M.P. and U.R. performed immunohistochemical staining. J.F. processed biological samples. E.K. and J.C. provided scientific advice. A.H. provided biological samples. C.S. supervised biological sample processing and provided neuropathological expertise. S.H. contributed biological samples, performed immunohistochemical staining, and advised on the interpretation of the findings. C.B. conceptualized and designed the study, prepared the original draft of the manuscript, and supervised the project. All authors contributed to critical revision of the manuscript for important intellectual content and approved the final version.

## Supporting information


**Supplementary Figure 1.**: Analysis of cortical cell distribution. (A) Median nearest neighbor distance (NND) analysis demonstrated (i) increased NND in CSF1R‐LE and (ii) a distinctly broader distribution of median values compared with controls (*p* = 0.0039), based on multiple regions per patient (*n*_CSF1R_ = 4, *n*_controls_ = 7). (B) Analysis of Δ median NND revealed a marked reduction in CSF1R‐LE (Δ = −10), indicating a strong deviation from a random spatial distribution (random distribution defined as Δ = 0.0). In contrast, control grey matter showed values close to randomness (Δ = +0.6; based on patient mean values, *p* = 0.051). (C), (D) Assessment of IBA1+ cells in exemplary lesion stage III sections, demonstrated a patchy cortical distribution of cells. (E)–(K) Marker analysis in cortical clusters revealed expression of P2RY12 in this cluster. In contrast, only weak expression of CD163, FTL, and CD68 was observed, while the homeostatic marker TMEM119 remained negative. Scale bars: C, D: 1 mm, E, F, H, I 100 μm, G, K 50 μm.

**Figure S1.** Myeloid cell marker densities by brain region. Quantifications of IBA1+ cells (A) and CD68+ cells (B) per brain region in patients and controls. Values are given as mean ± SEM of cells/mm^2^, calculated from patient‐level means.

**Supplementary Table S1.**: Case descriptions.

**Supplementary Table 2.**: List of antibodies used in the experiments.

**Supplementary Table 3.**: Characterization of Stage‐Specific Histopathological Features.

## Data Availability

The data that support the findings of this study are available on request from the corresponding author. The data are not publicly available due to privacy or ethical restrictions.
