# Supplementary material for: Spatial evolution of microglial phenotypes and iron accumulation in CSF1R‐associated leukoencephalopathy
Source: Brain Pathol. 2026 Nov 11:e70157. Online ahead of print. doi: 10.1111/bpa.70157 (PMC13754533; doi:10.1111/bpa.70157)
Supplement: Supplementary file 1 [file BPA-9999-e70157-s001.docx]

**Figure 1 Myeloid cell marker densities by brain region.** Quantifications of IBA1+ cells (A) and CD68+ cells (B) per brain region in patients and controls. Values are given as mean ± SEM of cells/mm², calculated from patient-level means.
