# Supplementary material for: Spatial evolution of microglial phenotypes and iron accumulation in CSF1R‐associated leukoencephalopathy
Source: Brain Pathol. 2026 Nov 11:e70157. Online ahead of print. doi: 10.1111/bpa.70157 (PMC13754533; doi:10.1111/bpa.70157)
Supplement: Supplementary file 2 [file BPA-9999-e70157-s002.docx]

Supplementary Table 3: Characterization of Stage-Specific Histopathological Features.

|  | **Stage** | | | |
| --- | --- | --- | --- | --- |
| **Marker** | **I** | **II** | **III** | **IV** |
|  |  |  |  |  |
| LFB PAS/HE | No loss of myelin fibers and no detectable axonal spheroids. | Initial appearance of axonal spheroids on H&E staining without visible reduction in fiber density. | Marked accumulation of axonal spheroids and numerous large, round cells containing dense intracellular material on H&E staining. | Pronounced loss of myelin fibers on LFB-PAS staining; axonal spheroids reduced secondary to extensive fiber loss. |
| MBP/ Neurofilament | High fiber density without visible accumulations or structural abnormalities. | Preserved fiber density without apparent reduction. | Reduced staining density of both NF and MBP, with NF loss preceding MBP reduction. | Severe reduction in fiber density. |
| APP | No evidence of axonal damage. | Focal accumulations of APP-positive deposits. | Extensive APP-positive deposits, including granular APP positivity within macrophages. | Decreased APP-positive deposits. |
