# Supplementary material for: Spatial evolution of microglial phenotypes and iron accumulation in CSF1R‐associated leukoencephalopathy
Source: Brain Pathol. 2026 Nov 11:e70157. Online ahead of print. doi: 10.1111/bpa.70157 (PMC13754533; doi:10.1111/bpa.70157)
Supplement: Supplementary file 3 [file BPA-9999-e70157-s003.docx]

Supplementary Table 2: List of antibodies used in the experiments.

| **Target** | **Host** | **dilution** | **company** | **Catalogue number** |
| --- | --- | --- | --- | --- |
| *Primary Antibodies* |  |  |  |  |
| Iba1 | rabbit | 1:500 | Synaptic Systems | 234 008 |
| Iba1 | goat | 1:500 | Abcam | ab5076 |
| CD68 | mouse | 1:300 | Dako | M0876 |
| KiM1P | mouse | 1:5000 | Radzun et al. 1991 |  |
| TMEM119 | rabbit | 1:200 | abcam | ab185333 |
| P2ry12 | rabbit | 1:1000 | NovusBio | NBP2-33870 |
| CD206 | mouse | 1:200 | abcam | ab117644 |
| CD163 | mouse | 1:200 | LeicaBiosystems | NCL-L-CD163 |
| Ferritin light chain (FTL) | mouse | 1:4000 | NovusBio | NBP2-54574UV |
| P22phox | rabbit | 1:1000 | abcam | ab75941 |
| Ferroportin | rabbit | 1:300 | NovusBio | NBP-73923 |
|  |  |  |  |  |
| TPPP |  |  |  |  |
| BCAS1 | mouse | 1:400 | SantaCruz | sc-136342 |
|  |  |  |  |  |
| MBP | chicken | 1:500 | Invitrogen | PA1-10008 |
| Neurofilament (NF) | mouse | 1:100 | Dako | M0762 |
| APP | mouse | 1:800 | Millipore | MAB348 |
|  |  |  |  |  |
| *Secondary Antibodies* |  |  |  |  |
| Biotinylated Anti-Rabbit | donkey | 1:100 | Jackson | AB_2340785 |
| Biotinylated Anti-Mouse | donkey | 1:100 | Jackson | AB_2340593 |
|  |  |  |  |  |
| Anti-goat 647 | donkey | 1:500 | Invitrogen | A21447 |
| Anti-rabbit 568 | donkey | 1:500 | Invitrogen | A10042 |
