# Supplementary material for: Spatial evolution of microglial phenotypes and iron accumulation in CSF1R‐associated leukoencephalopathy
Source: Brain Pathol. 2026 Nov 11:e70157. Online ahead of print. doi: 10.1111/bpa.70157 (PMC13754533; doi:10.1111/bpa.70157)
Supplement: Supplementary file 4 [file BPA-9999-e70157-s004.docx]

Supplementary Figure 1

**Supplementary Figure 1: Analysis of cortical cell distribution.** A: Median nearest neighbor distance (NND) analysis demonstrated (i) increased NND in CSF1R-LE and (ii) a distinctly broader distribution of median values compared with controls (p = 0.0039, based on multiple regions per patient (n_CSF1R_ = 4, n_controls_ = 7). B: Analysis of Δ median NND revealed a marked reduction in CSF1R-LE (Δ = −10), indicating a strong deviation from a random spatial distribution (random distribution defined as Δ = 0.0). In contrast, control grey matter showed values close to randomness (Δ = +0.6; based on patient mean values, p= 0.051). C-D: Assessment of IBA1+ cells in exemplary lesion stage III sections, demonstrated a patchy cortical distribution of cells. E-K: Marker analysis in cortical clusters revealed expression of P2RY12 in this cluster. In contrast, only weak expression of CD163, FTL, and CD68 was observed, while the homeostatic marker TMEM119 remained negative. Scale bars:C, D: 1mm, E, F, H, I 100µm, G, K 50µm.
