# Supplementary material for: Spatial evolution of microglial phenotypes and iron accumulation in CSF1R‐associated leukoencephalopathy
Source: Brain Pathol. 2026 Nov 11:e70157. Online ahead of print. doi: 10.1111/bpa.70157 (PMC13754533; doi:10.1111/bpa.70157)
Supplement: Supplementary file 5 [file BPA-9999-e70157-s005.docx]

Supplementary Table S1: Case descriptions.

|  | **sex** | **Age at death** | **Cause of death** | **Postmortem interval**  **(d)** | **Age at symptom onset** | **Initial symptoms** | **Genetic variant** |
| --- | --- | --- | --- | --- | --- | --- | --- |
| *CSF1R-LE* |  |  |  |  |  |  |  |
| #1 | f | 46 | Vegetative state | 1 | 43 | Cognitive decline, behavioral  abnormalities, lack of drive,  motor deficits, imbalance,  repetitive falling | c.2467G>A; p.(Ala823Thr) |
| #2 | m | 52 | Pneumonia | 1 | 49 | Cognitive decline, behavioral  abnormalities, Apraxia | c.2563C>T; p.(Pro85Ser) |
| #3 | f | 66 | Myocardial infarction | 1 | 60 | Speaking impairment, gait  disorder, imbalance | c.2381T>C; p.(Ile794Thr) |
| #4 | NA | NA | NA | NA | NA | NA | NA |
| #5 | m | 58 | Pneumonia | NA | 51 | Personality change, seizures, cognitive decline | NA |
|  |  |  |  |  |  |  |  |
| *Control cohort* |  |  |  |  |  |  |  |
| #1C | m | 52 | Respiratory failure | 2 |  |  |  |
| #2C | m | 71 | Colitis | 2 |  |  |  |
| #3C | f | 60 | Mycosis | 2 |  |  |  |
| #4C | m | 68 | Colon ischemia | 2 |  |  |  |
| #5C | f | 69 | Heart failure | 2 |  |  |  |
| #6C | m | 74 | Pneumonia | 2 |  |  |  |
| #7C | f | 61 | Heart failure | 2 |  |  |  |
| #8C | m | 68 | Liver failure | 2 |  |  |  |
| #9C | m | 72 | Pulmonary embolism | 1 |  |  |  |
| #10C | m | 55 | myocardial infarction | 3 |  |  |  |
| #11C | m | 57 | Pulmonary embolism | 4 |  |  |  |
| #12C | w | 60 | myocardial infarction | 5 |  |  |  |
| #13C | w | 62 | Aortic dissection | 1 |  |  |  |
| #14C | w | 63 | Aortic dissection | 5 |  |  |  |
| #15C | m | 56 | myocardial infarction | 3 |  |  |  |
